# Evaluation of the in vitro skin permeation of antiviral drugs from penciclovir 1% cream and acyclovir 5% cream used to treat herpes simplex virus infection

**DOI:** 10.1186/1471-5945-9-3

**Published:** 2009-04-02

**Authors:** Nathalie Hasler-Nguyen, Donald Shelton, Gilbert Ponard, Marlene Bader, Martina Schaffrik, Pascal Mallefet

**Affiliations:** 1 Department of Preclinical Development, Novartis Consumer Health, Nyon, Switzerland; 2 Department of Medical Affairs, Novartis Consumer Health, Munich, Germany; 3 Department of Global Medical Affairs, Novartis Consumer Health, Nyon, Switzerland

## Abstract

**Background:**

Herpes simplex virus infection (HSV) is a common and ubiquitous infection of the skin which causes mucocutaneous lesions called cold sores (herpes labialis) or fever blisters. It is estimated that approximately 80% of the population worldwide are carriers of the Herpes simplex virus, approximately 40% suffer from recurrent recurrent infections. This study evaluates the *in vitro *skin permeation and penetration of penciclovir and acyclovir from commercialized creams for the treatment of herpes labialis (cold sores), using non viable excised human abdominal skin samples, which were exposed to 5 mg/cm^2 ^of acyclovir 5% cream or penciclovir 1% cream.

**Methods:**

After 24 h of cream application, excess cream was washed off and layers of stratum corneum were removed by successive tape stripping. Amounts of active ingredients having penetrated through the skin were measured, as well as the amounts in the washed-off cream, in skin strips and creams remaining in the skin. Molecular modelling was used to evaluate physico-chemical differences between the drugs. Western blot analysis enabled to determine whether the marker of basal cells keratin 5 could be detected in the various tape strips.

**Results:**

Application of penciclovir 1% cream yielded higher concentration of drug in the deeper layers of the epidermis as well as a higher drug flux through the skin. Molecular modelling showed two higher hydrophobic moieties for acyclovir. Presence of the basal cell marker keratin 5 was underscored in the deeper tape strips from the skin, giving evidence that both drugs can reach their target cells.

**Conclusion:**

Penciclovir 1% cream has the tendency to facilitate the diffusion of the drug through the stratum corneum into the deeper epidermis layers, in which it could reach the target basal cells at effective therapeutical concentration. The small difference in the surface properties between both molecules might also contribute to favour the passage of penciclovir through the epidermis into the deeper basal cells.

## Background

Herpes simplex virus infection (HSV) is a common and ubiquitous infection of the skin which causes mucocutaneous lesions called cold sores (herpes labialis) or fever blisters. The vast majority of cold sores are due to herpes simplex virus type 1 (HSV-1). It is estimated that approximately 80% of the population worldwide are carriers of the Herpes simplex virus, approximately 40% suffer from recurrent infections [1,2]. About 1% of sufferers have frequent, i.e. monthly outbreaks of the latent herpes infection. These infections last for 4 to 10 days and can extend up to 30 days in immunocompromised patients where lesions may develop extensive necrosis [1]. Topical treatment with antiviral drugs like acyclovir and penciclovir is effective in shortening lesion duration and in relieving pain, as has been shown in large randomized, double-blind, vehicle controlled multi-centre clinical trials [1-4].

**Figure 1:**
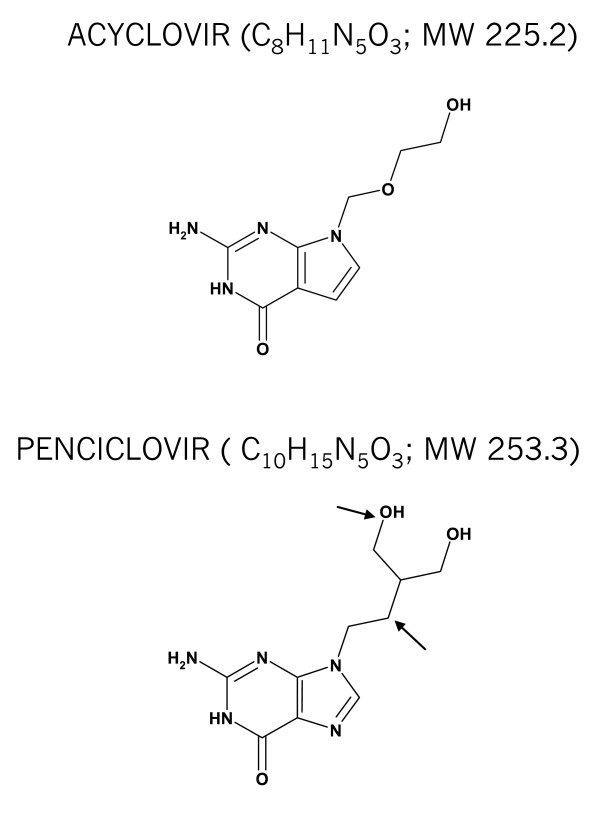
**Chemical structures of acyclovir and penciclovir**. Differences are indicated by arrows in the penciclovir structure.

For best effect, antiviral drugs should be able to reach therapeutic concentrations in basal epidermal cells, which are the initial portal of entry to the virus spreading [5,6]. Consequently, skin absorption is one of the most critical factors for successful therapy with a topical formulation for the treatment of herpes labialis. The route of drug penetration across the stratum corneum, which is the main rate limiting barrier to skin absorption [7], depends on the properties of the respective drugs. Penciclovir has an additional hydroxyl group when compared to acyclovir (Figure 1), which became available as a generic drug in the mid 1990s when its analogue penciclovir was launched [8].

As the permeability properties of the stratum corneum remain unchanged after removal from the body, a good correlation has been observed between *in vivo *and *in vitro *experiments with the same drug [9-11]. Thus, *in vitro *diffusion through non viable human skin is a convenient experimental tool to explore permeation characteristics of a drug from its topical formulation.

The present study compared the *in vitro *skin distribution and permeation of two commercialized creams containing 1% penciclovir or 5% acyclovir, both containing approximately 40% propylene glycol. *In vitro *skin permeation was assessed using human skin mounted in Franz-type diffusion cells [12]. Following topical application of 5 mg/cm^2 ^cream on excised human skin, drug permeation into the receptor fluid of the diffusion chamber was measured after 24 h. At the end of the experiment drug levels in the remaining skin were determined after removal of the stratum corneum by tape stripping. Additionally, physico-chemical differences between the drugs were investigated by molecular modelling as this is a factor affecting dermal absorption [13].

## Methods

### Products

Penciclovir cream 1% (10 mg/g penciclovir, Fenivir, Novartis Consumer Health, Switzerland) was provided *in house *and acyclovir cream 5% (50 mg/g acyclovir; Zovirax, GlaxoSmithKline, United Kingdom) was purchased from a Swiss pharmacy.

Penciclovir was provided *in house *and acyclovir was bought from Sigma Chemicals (Switzerland).

### Skin donor

After securing the appropriate consent the ethical committee of the International Institute for the Advancement of Medicine (IIAM; United States) reviewed and approved our application for request of human tissue to be used in this study.

Full thickness human abdominal skin from 6 donors, which was taken at autopsy, was provided cryopreserved by IIAM. The skin was maintained frozen at -80°C. Prior to use, the skin was thawed and the subcutaneous tissue was carefully removed. The skin was dermatomed to 500 μm using a Wagner dermatome (model GB-231 Aesculap, Germany), which enabled us to obtain split thickness tissue constituted of the stratum corneum (10–20 μm), the epidermis (100 μm) and part of the dermis (1200 μm) [14,15].

### Test of skin integrity

The permeation of tritiated water was evaluated to determine the skin integrity as described by Bronaugh [16]. Briefly, tritiated water (2.7 μCi/ml) was applied to the skin surface. After 30 min, the radiolabelled water was removed from the skin with cotton-wool tips. The receptor phase (2 ml) was taken in order to measure the amount of tritiated water (%) which permeated across the skin, using a liquid scintillation counter. Formulations were tested on skin samples having similar tritiated water permeation values. Less than 1% of the applied dose of tritiated water permeated through the skin.

### Skin permeation

Skin permeation is the diffusion of the drug across the skin layer into the receptor phase which represents blood vessels. It was measured using a static Franz diffusion cell of 1.75 cm^2 ^for each skin sample exposed to the product to be tested at 5 mg/cm^2 ^simulating in use conditions and in accordance to the test guideline OECD 428 [17].

Thawed human dermatomed skin samples were mounted horizontally on the Franz cells, dermis side down. The Franz cells were connected to a circulating water bath at 37°C, which yielded a tissue temperature of 32°C, comparable to the physiological temperature of the skin surface. The receptor phase of PBS pH 7.4 (phosphate buffered saline; 7.58 g/L Na_2_HPO_4_, 1.62 g/L NaH_2_PO_4 _and 4.4 g/L NaCl) contained within each diffusion cell (approximately 8 ml) was mixed using a magnetic stirring device to ensure appropriate homogenization of the released drug in the acceptor phase throughout the experiment. Samples of the receptor phase were collected at 24 h.

### Skin penetration

The skin penetration was determined by measuring the amount of drug residing in the various skin layers.

At the end of the 24 h after application skin samples were washed off with soapy water and cotton-wool tips. The top layer of the stratum corneum was removed with adhesive tape (3 M Scotch n° 550) and analyzed separately. Additional layers of the stratum corneum were removed by up to 12 consecutive adhesive tape strips.

Following collection of all strips, the first and the second strips were put into separate vials containing 10 ml of water. Strips 3 up to 7 were pooled in the same vial containing 20 ml of water. The remaining stripped skin was minced and placed into a vial containing 15 ml water. Mixtures were stirred overnight to ensure adequate extraction of the drug from the tape and the skin.

### Sample analysis

Penciclovir and acyclovir content in the various samples was measured by high performance liquid chromatography (Agilent HP 1100) on a Waters Spherisorb^® ^5 μm ODS2 (4.6 × 250 mm Analytical Cartridge PSS839540) phase column at 35°C using a 1 ml flow rate of the mobile phase of a mixture of methanol and 0.1 M pH 6.0 ammonium acetate buffer 1:10 (v:v) with UV detection at 254 nm. The sample was directly injected at a volume of 50 μl. Drug concentrations were determined from penciclovir or acyclovir standard curves between 10 and 50 ng/ml generated with the pure compounds solubilized in PBS at pH 7.4, which was the solvent used in the receptor phase. Maximal soluble concentration in this solvent was 0.9 mg/ml for penciclovir and 0.7 mg/ml for acyclovir. The retention time was respectively 6.7 min for penciclovir and 5.1 min for acyclovir. The limit of quantitation (LOQ) was 7 ng/ml for both drugs.

### Molecular modelling

The characteristics of the penciclovir and acyclovir chemical structures were evaluated with an *in house *web based CHEMINFORMATICS system using the CORINA program. Generation and display of molecular surface properties enabled us to reveal those parts of the molecules which are involved in hydrophobic or hydrophilic interactions [18].

### Western blot analysis

The skin sample from one donor was washed off with soapy water and cotton-wool tips 24 h after the cream application. The top layer of the stratum corneum was removed with adhesive tape (3 M Scotch n° 550) and analyzed separately. Additional layers of the stratum corneum were removed by up to 12 consecutive adhesive tape strips. Following collection of all strips, the first and the second strips were put into separate vials. Then pools of 3 to 4 strips were put in the same vial and the last strip was placed separately in another vial. A volume of 250 μl/tape strip of ice-cold lysis buffer (20 mM Tris-HCl; 2 mM EGTA; 2 mM EDTA; 30 mM NaF; 30 mM Na_4_O_7_P_2_; 2 mM Na_3_VO_4_; 1 mM [4-(2-Aminoethyl)benzenesulfonylfluoride] (AEBSF); 10 μg/ml leupeptin; 4 μg/ml aprotinin; 1% Triton X-100; pH 7.4) was added in the vial, which was left for 10 min on ice and then vortexed. Supernatants were collected and stored at -20°C overnight. Protein concentrations were measured by the BCA protein assay (Pierce).

Ten μl of protein samples corresponding to 3 μg of protein from tape strips lysat were separated by 10% SDS-PAGE and transferred to polyvinylidene difluoride membranes (Immobilon-P, Millipore, Bedford, MA). Membranes were blocked by incubation with Trisbuffered saline (50 mM Tris, 150 mM NaCl) containing 0.2% (v/v) Nonidet P-40 and 5% (w/v) nonfat dry milk for 30 min at room temperature. Membranes were probed overnight at 4°C with a monoclonal mouse anti-human Keratin 5 (Millipore) antibody (1:20,000) and then with secondary horseradish peroxidase-conjugated goat anti-human IgG (1:20,000) (Transduction Laboratories, Lexington, KY) for 1 h at room temperature. The membranes were washed three times with Tris-buffered saline containing 0.2% (v/v) Nonidet P-40, and the antigen-antibody complexes were detected by the Super Signal Substrate method (Pierce). Identified protein bands were detected using a video densitometer and ImageQuant software (Molecular Dynamics, Sunnyvale, CA).

### Statistical analysis

Unpaired Student t-test were performed to define a P value between the pencyclovir cream and the acyclovir cream. A P value of less than 0.05 was considered statistically significant.

## Results

After the 24 h skin exposure time creams were washed off from the skin and the acyclovir cream had 6-fold more drug remaining in the washed-off cream than the penciclovir cream, with 245 μg/cm^2 ^versus 40 μg/cm^2^, respectively (not shown). These values represent the amount of unabsorbed drug.

**Figure 2:**
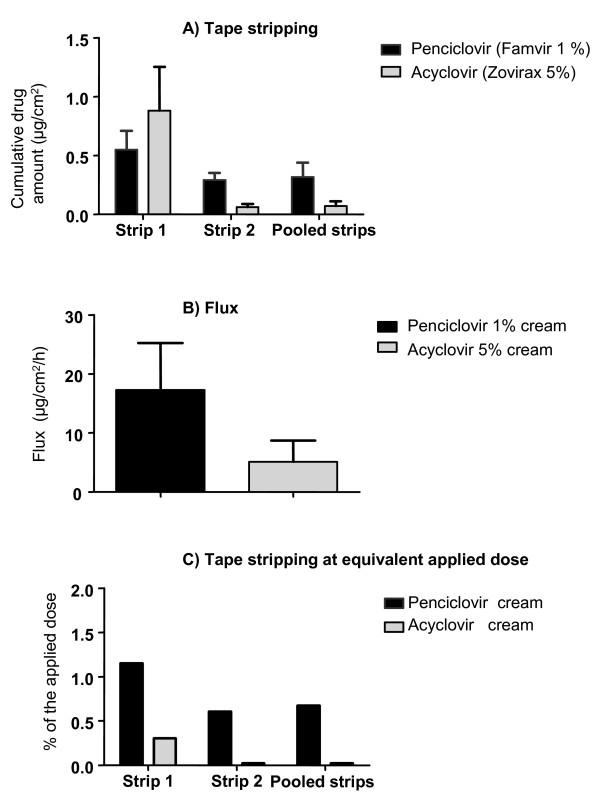
**Comparison of drug concentration found in the tape stripping (A) and expressed as skin flux (B) or adjusted to the applied dose (C) after application of Penciclovir 1% cream and Acyclovir 5% cream on excised human skin after 24 h**. Values represent mean from 11 replicates with standard error of the mean.

Cumulative amounts of each drug that had penetrated into the skin are presented in Figure 2A. When acyclovir was applied on the skin as 5% cream, the highest concentration of acyclovir was found in strip 1 (0.88 μg/cm^2^) while in the deeper successive strip (strip 2) the drug was found at significantly lower concentration than penciclovir from the 1% cream (Table 1; P = 0.002). In the pooled strip 4.5 fold less acyclovir was found than for penciclovir. This difference approached statistical significance (P = 0.07). Penciclovir from the 1% cream was found in the first strip at 0.55 μg/cm^2 ^while analysis of deeper successive strips revealed 0.29 μg/cm^2 ^penciclovir in the 2^nd ^strip and 0.32 μg/cm^2 ^in the pooled strip (from 3 strips up to 7 strips) (Figure 2A). The total concentration of drug found in the pooled strips might include the entire epidermis in accordance with the finding that after 3 h of experiment duration the epidermis peels off with the stratum corneum [19].

**Table 1:** Comparison of the amount of antiviral drug found in the various skin compartments.

	**Penciclovir cream (P)**		**Acyclovir cream (A)**		**Ratio (P/A)**	**Statistics**
μg/cm^2^	Mean	s.e.m	Mean	s.e.m		P value

Strip 1	0.55	0.16	0.88	0.3	0.6	0.42

Strip 2	0.29	0.06	0.06	0.03	4.9	0.002

Pooled Strips	0.32	0.12	0.07	0.04	4.5	0.07

**Epidermis**	**1.15**	**0.35**	**1.01**	**0.04**	**1.1**	**0.32**

**Dermis **(residual stripped skin)	**0.10**	**0.04**	**0.07**	**0.04**	**1.4**	**0.63**

**Permeation through the skin **(receptor phase)	**0.41**	**0.19**	**0.12**	**0.10**	**3.4**	**0.18**

	**Penciclovir cream (P)**		**Acyclovir cream (A)**		**Ratio** **(P/A)**	**Statistics**

% applied dose	Mean	s.e.m	Mean	s.e.m		P value

Strip 1	1.15	0.41	0.30	0.11	3.8	0.06

Strip 2	0.61	0.16	0.02	0.01	29.1	0.002

Pooled Strips	0.67	0.31	0.02	0.01	32.3	0.04

**Epidermis**	**2.4**	**0.9**	**0.3**	**0.1**	**7.0**	**0.3**

**Dermis **(residual stripped skin)	**0.17**	**0.07**	**0.02**	**0.01**	**9.1**	**0.06**

**Permeation through the skin **(receptor phase)	**0.68**	**0.33**	**0.04**	**0.03**	**16.7**	**0.06**

Values represent mean from 11 replicates with standard error of the mean (s.e.m).

*In vitro *human skin percutaneous absorption of penciclovir and acyclovir from creams as cumulative permeation and when adjusted to the applied dose.

After 24 h the amount of penciclovir permeated through the skin from the 1% cream was 3.4 fold more than acyclovir from the 5% cream (0.41 vs. 0.12 μg/cm^2^) (Table 1), leading to a flux of respectively 17 ng/cm^2^/h and 5 ng/cm^2^/h as depicted in Figure 2B.

When the results were compare at equivalent dose 16.7 fold more penciclovir from the 1% cream permeated through the skin than acyclovir, which was mainly limited to the superficial skin layer. This difference show a trends toward statistical difference (P = 0.06; Figure 1C and Table 1). Significantly more penciclovir were detected in deeper strips such as strip 2 (P = 0.002) and pooled strips (P = 0.04). The difference between penciclovir and acyclovir found in the dermis were close to statistical significance (P = 0.06; Figure 1C and Table 1). Permeation through the excised human skin reached 3.3% of the applied dose for penciclovir and 0.2% for acyclovir (Table 1).

**Figure 3:**
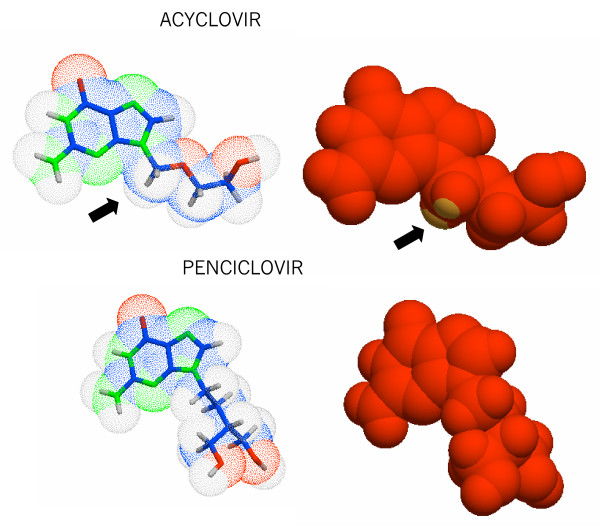
**Display of the molecular surface properties of acyclovir and penciclovir as determined by the molecular modelling**. This reveals parts of the molecule which participate to hydrophobic or electrostatic interactions, which might result in bioavailability difference. Dark areas indicate hydrophilic areas and lighter areas are hydrophobic moieties, indicated by an arrow.

Molecular modelling enables to generate and display the molecular surface properties such as electrostatic potential, lipophilicity potential or polar surface area revealing parts of the molecule which are involved in hydrophobic or electrostatic interactions or which may cause bioavailability difference. Thus this chemical modelling software was used to compare acyclovir and penciclovir which are known as hydrophilic drugs. As shown in Figure 3 the surface charge distribution is very similar but the hydrophobicity distribution showed two areas of higher hydrophobic moieties for acyclovir.

## Discussion

Measurement of *in vitro *permeation across the split thickness of human skin enables the evaluation of the passive diffusion of the molecule into and across the skin to a solute reservoir, which simulates the capillaries of the dermis. Several factors influence the skin permeation among which are the release of the drug from the formulation, its penetration into the stratum corneum layers (10–20 μm) and its diffusion through the stratum corneum into the various epidermis layers (100 μm) and the dermis (1200 μm) to reach the circulation, as simulated by the fluid in the reservoir.

When compared at an equivalent dose penciclovir from the cream has the trend to have a higher transcutaneous delivery than acyclovir. These results indicate a more effective release of penciclovir from the cream and across the stratum corneum. Both formulations contain approximately 40% of propylene glycol (Acyclovir, 5% Zovirax contains 40% and penciclovir 1% Pencivir contains 43%) which is widely use as a co-solvent and penetration enhancer [20]. This fatty alcohol, which diffuses well through the stratum corneum [20] might alter the properties of this membrane leading to improved drug distribution especially for penciclovir, while further permeation into the receptor fluid might be impaired for acyclovir. This difference between these structurally related polar molecules might be explained by the formulation itself together with the presence of hydrophobic region in the acyclovir structure as determined by the molecular modelling. This moiety may interact with hydrophobic structure in the stratum corneum preventing further penetration. It has been reported that increased lipophilicity is used to reduce systemic absorption, which was reported for the corticosteroids [21]. The hydrophilic feature of penciclovir as shown in Figure 3 might not react with hydrophobic structures, thus allowing penciclovir to be freely "drawn" by the solvent between the corneocytes of the stratum corneum within the hydrophilic region, constituting the paracellular pathway [13,22].

**Figure 4:**
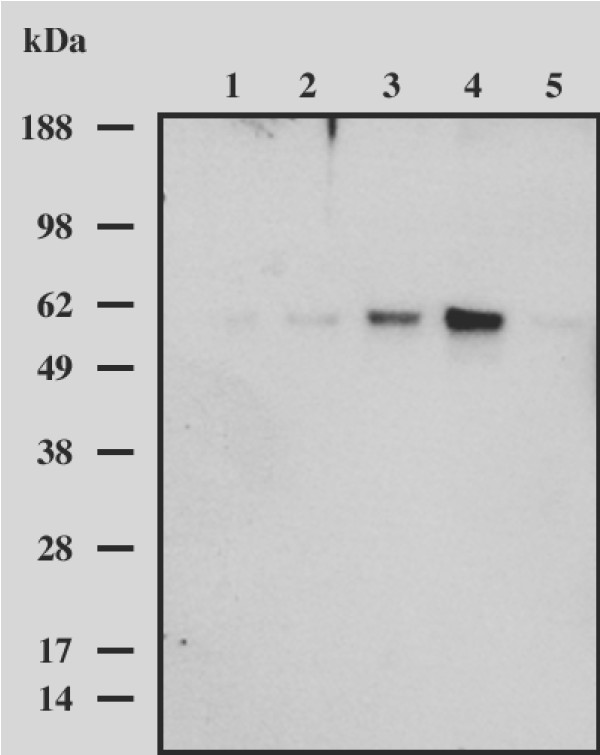
**Keratin 5 in tape strips from excised human skin treated with penciclovir 1% cream was detected by Western blot analysis**. For the Strip 1 in lane 1, the Strip 2 in lane 2 and the last Strip in lane 5 the signal of the 60 kD band was faint when compared to the pooled strips in lane 3 and 4.

The stripping technique enables measurement of drug concentration in the stratum corneum by repeated application of adhesive tape to the skin sample generally between 10 to 15 times [14]. After a 3 h duration of this experimental technique it has been reported that the stratum corneum peels readily from the dermis including all remaining epidermal layers [15,19]. It was questionable whether under our experimental conditions, in which the tape stripping was done 24 h after the product application basal cells could be found in the tape strips. Knowing that keratin 5 is specifically expressed in the basal cell of the epidermis [23] a western blot analysis using a monoclonal antibody against this protein was performed. As shown in Figure 4 this technique produced a narrow, unique 60 kDa band in samples of pooled tape strips (on lane 3 and 4) and only very faint bands of similar size from samples of tape strips 1, 2 and the last strip (on lane 5). This band corresponds to the known molecular weight of keratin 5, which is 58 kDa.

These results give evidence that basal cells were removed during the tape stripping and were found mostly in the deeper epidermal layer. Thus, concentrations per area of penciclovir in pooled strips were converted into concentration by volume with an estimate of epidermal depth of 100 μm [14], which yielded a 60 fold higher concentration than the *in vitro *inhibitory effect on HSV-1 with IC_50 _of 0.5–0.8 μg/ml as reported by Weinberg [24]. This suggests penciclovir might be highly efficient in reducing the viral load in the infected cells of the basal epidermal layers when compared to the surface of epidermis represented by the stratum corneum.

It is questionable whether the observed concentration differences found between the two drugs at the different skin layers might be due to the different intracellular half lives of acyclovir (0.7–1 h) and penciclovir (10–20 h) [25-27]. As the excised skin was frozen before use this tissue might not be viable lacking metabolism and enzymatic activity [28]. Consequently, this excludes under experimental conditions of the present study that viral thymidine kinase could be active and could phosphorylate both drugs which would be retained in cells [25]. In the future it could be of interest to perform the study with fresh and viable excised skin infected or not by HSV-1 to evaluate the contribution of the viral enzymatic activity on the drug bioavailability into the deeper epidermal layers where the target cells are present.

## Conclusion

In this *in vitro *model the passive diffusion of two hydrophilic drugs from two comparable formulations were studied showing both creams delivered the drug in deeper epidermal skin layers where target basal cells were localized as determined by the expression of the specific keratin 5 protein. Molecular modelling enabled for the first time to reveal difference between acyclovir and penciclovir on the surface properties. This observation might support that penciclovir has the tendency to have a higher paracellular passage through the stratum corneum resulting in a tendency to reach higher concentration in deeper epidermal layers.

As permeability properties of the stratum corneum remained unchanged in non viable human skin the findings of the present study might be extrapolated to in use condition. By applying the cream at the prodormal stage, when skin appears undamaged it can be postulated the drug would be delivered at the level of basal cells where the HSV-1 virus could be found in case of cold sore infection.

## Competing interests

The authors declare that they have no competing interests.

## Authors' contributions

NHN contributed to the analysis, interpreted results and drafted manuscript. DS, GP and MB carried out the experimental part and contributed to result analysis. MS and PM have made substantial contribution to drafting and revising the manuscript critically. All authors read and approved the final manuscript.

## Pre-publication history

The pre-publication history for this paper can be accessed here:


